# Single vagus nerve stimulation reduces early postprandial C-peptide levels but not other hormones or postprandial metabolism

**DOI:** 10.1007/s10067-017-3618-5

**Published:** 2017-04-08

**Authors:** M. W. Tang, F. S. van Nierop, F. A. Koopman, H. M. Eggink, D. M. Gerlag, M. W. Chan, R. Zitnik, F. M. Vaz, J. A. Romijn, P. P. Tak, M. R. Soeters

**Affiliations:** 1 Department of Clinical Immunology & Rheumatology, Amsterdam Rheumatology and Immunology Centre, Academic Medical Centre, University of Amsterdam, Room F4-105, PO Box 22700, 1100 DE Amsterdam, The Netherlands; 2 Department of Experimental Immunology, Academic Medical Centre, University of Amsterdam, Amsterdam, The Netherlands; 3 Department of Endocrinology and Metabolism, Academic Medical Centre, University of Amsterdam, Amsterdam, The Netherlands; 4 Currently also Clinical Unit Cambridge, GlaxoSmithKline, Cambridge, UK; 5 SetPoint Medical Corporation, Valencia, CA USA; 6 Laboratory of Genetic Metabolic Disease, Department of clinical chemistry, Academic Medical Centre, University of Amsterdam, Amsterdam, The Netherlands; 7 Department of Internal Medicine, Academic Medical Centre, University of Amsterdam, Amsterdam, The Netherlands; 8 Currently also GlaxoSmithKline, Stevenage, UK; 9 University of Cambridge, Cambridge, UK; 10 Ghent University, Ghent, Belgium

**Keywords:** Brain, Clinical trials and methods, Endocrine, Gastrointestinal, Hormones, Metabolic disease, Metabolomics, Rheumatoid arthritis, Study design

## Abstract

A recent study in rheumatoid arthritis (RA) patients using electrical vagus nerve stimulation (VNS) to activate the inflammatory reflex has shown promising effects on disease activity. Innervation by the autonomic nerve system might be involved in the regulation of many endocrine and metabolic processes and could therefore theoretically lead to unwanted side effects. Possible effects of VNS on secretion of hormones are currently unknown. Therefore, we evaluated the effects of a single VNS on plasma levels of pituitary hormones and parameters of postprandial metabolism. Six female patients with RA were studied twice in balanced assignment (crossover design) to either VNS or no stimulation. The patients selected for this substudy had been on VNS therapy daily for at least 3 months and at maximum of 24 months. We compared 10-, 20-, and 30-min poststimulus levels to baseline levels, and a 4-h mixed meal test was performed 30 min after VNS. We also determined energy expenditure (EE) by indirect calorimetry before and after VNS. VNS did not affect pituitary hormones (growth hormone, thyroid stimulating hormone, adrenocorticotropic hormone, prolactin, follicle-stimulating hormone, and luteinizing hormone), postprandial metabolism, or EE. Of note, VNS reduced early postprandial insulin secretion, but not AUC of postprandial plasma insulin levels. Cortisol and catecholamine levels in serum did not change significantly. Short stimulation of vagal activity by VNS reduces early postprandial insulin secretion, but not other hormone levels and postprandial response. This suggests VNS as a safe treatment for RA patients.

## Introduction

The autonomous nervous system and the immune system interact in both directions via neurotransmitters, cytokines, and hormones [1, 2]. The parasympathetic nervous system, which consists mainly of the vagus nerve, includes afferent nerves that signal the state of the viscera to the central nervous system [3, 4]. The so-called inflammatory reflex represents a reflex circuit defined by signals that travel in the vagus nerve to inhibit monocyte and macrophage production of tumor necrosis factor (TNF) and other cytokines [5]. Of interest, recent prospective observational studies indicate that impaired constitutive vagus nerve activity precedes the development of clinically manifest rheumatoid arthritis (RA) [6]. Activation of this pathway through electrical vagus nerve stimulation (VNS) could be a feasible and effective treatment in reducing pathological systemic inflammation in RA and other inflammatory diseases like Crohn’s disease [7, 8]. Recently, it has been shown that free fatty acids, norepinephrine, and pancreatic polypeptide levels are increased in individuals at risk for development of RA and RA patients, compared to healthy individuals [9]. These findings could suggest the hypothesis that VNS could influence those peptides and therefore also (the development of) RA. There is extensive experience with VNS as a long-term treatment in patients with refractory epilepsy and depression [10, 11]. Recently, a phase 2, open-label multicentre study showed that VNS in RA patients significantly inhibited TNF production and improved disease activity [12]. In this study, the left vagus nerve was isolated within the sheath between the carotid artery and the internal jugular vein and a VNS lead with three helical coiled cuffs was placed around the vagus nerve. With this approach, we cannot exclude the possibility that VNS has effects in other vagal targets, such as the pituitary gland and the central nervous system.

Pituitary hormone levels are known to be partially under vagal control, as demonstrated by a significant increase in prolactin (PRL) levels after bilateral vagotomy in rats [13]. In lactating rats, vagotomy blunts the suckling-induced increase of PRL [14]. There are conflicting data on the effect of VNS on the hypothalamic-pituitary-adrenal (HPA) axis in animal models [15, 16]. However, there are currently no data on the effects of VNS on PRL levels in humans. One study has suggested a reduction in the adrenocorticotropic hormone (ACTH) response upon corticotropin-releasing hormone (CRH) challenge after short-term VNS (during 2 weeks) in patients with chronic depression [17].

The vagus nerve also regulates metabolic activity through visceral afferent and efferent fibers. Afferent signals from hormones, chemokines, and nutrients inform the brain on feeding and energy status. This could indirectly influence thermoregulation by modulation of CRH release from the hypothalamus [18]. Selective efferent VNS increased serum insulin levels, although afferent VNS caused a sustained increase in glucose in rats [19]. Parasympathetic hepatic denervation in obese Zucker rats leads to increased cholesterol levels. In the only available human data, VNS significantly increased energy expenditure (EE) in patients with refractory epilepsy [20, 21].

The objective of this pilot study was to assess the acute response in plasma hormone and nutrient concentrations after a single VNS, as well as the postprandial metabolic response in RA patients on daily VNS treatment.

## Subjects and methods

### Patients

Six female patients with RA were enrolled. Prior to enrollment in this substudy, patients first participated in a core study (NCT01552941) in which they were implanted with a VNS device to assess safety and efficacy of daily VNS on RA outcomes [22]. These patients also participate in a long-term extension of the core study (NCT01552538). Patient participation in the current substudy was optional.

Subjects were included between October 2013 and March 2014 in this substudy. The study patients were included at the Department of Clinical Immunology and Rheumatology of the Academic Medical Center, Amsterdam, the Netherlands. Enrolled RA patients fulfilled the 2010 ACR/EULAR classification criteria for RA [23, 24]. Five patients were using nonsteroidal anti-inflammatory drugs (NSAIDs). All RA patients were treated with disease-modifying anti-rheumatic drugs (DMARDs), four patients used corticosteroids till the maximum dose of ≤7.5 mg/day, and one patient used biologicals. The core study, the extension, and the current substudy were separately approved by the institutional review board of the AMC and performed according to the principles of the Declaration of Helsinki. All study subjects gave written informed consent for all study protocols separately.

### Study design

Patients were studied twice with balanced assignment (crossover design) between VNS and no stimulation during the course of their regular daily VNS treatment. The study days were separated by ≤2 weeks to minimize influence of changes in disease activity. Patients were instructed not to activate the VNS 1 day prior to the study visits. Additionally, they were instructed not to use alcohol (2 days prior to study visit) and tobacco products or to drink coffee/tea or ingest any other caffeine-containing products within 1 day prior to study visit.

On study days, patients were admitted to the metabolic unit at 08:00 h after an overnight fast. A catheter was inserted into a hand/arm vein and kept in a thermoregulated (60 °C) clear plastic box for sampling of arterialized venous blood. Saline was used to keep the catheter patent. From *t* = 0 (09:00 h), three blood samples were drawn at 10-min intervals for determination of baseline hormone levels. At *t* = 30 min (09:30 h), patients used magnet actuation to activate the VNS once or not (depending on study day) after which three blood samples were drawn at 10-min intervals. At *t* = ‘0 min (10:30 h), a liquid mixed meal (Nutridrink Compact, Nutricia, Zoetermeer, the Netherlands) containing 25% of daily EE (estimated using the Harrison Benedict equation) was given, after which blood was drawn at 30-min intervals at ’30, ’60, ’90, ’120, ’150, ’180, ’210 and ’240 min.

### VNS and device parameters

The device parameters define modulations of vagus nerve firing and elicit the neurostimulated inflammatory reflex. The device parameters for each patient, including pulse frequency, pulse duration and output current, and VNS stimulation are detailed in Table 1. The output current was set at the maximum level tolerated for each patient with a maximum limit of 2.0 mA. For all patients, the signal frequency was 10 Hz with a pulse duration of 250 μs. The frequency of daily stimulations was between one time and eight times daily. The patients selected for this substudy had used VNS for at least 3 months and at maximum of 24 months. Confirmation of VNS activity after magnet actuation was obtained by noting physical signs of vagal nerve activity, such as transient hoarseness, coughing, and palpitations.

**Table 1:** Device parameters per patient

Patient ID	Pulse frequency (Hz)	Pulse width (μs)	Output current (mA)	Stimulation time (s)	Frequency of VNS per day (times)	Prior duration of VNS use (months)
1	10	250	2.00	60	1	5
2	10	250	1.25	60	6	5
3	10	250	1.25	60	8	6
4	10	250	1.75	60	6	24
5	10	250	2.00	60	6	7
6	10	250	1.50	60	8	3

### EE and HRV

Oxygen consumption and carbon dioxide production were measured for 20 min in a supine position using a ventilated hood system (Vmax Encore 29; SensorMedics, Anaheim, CA). EE was calculated from oxygen consumption and carbon dioxide production as reported previously [25]. EE and eardrum temperature were measured at baseline (*t* = −30 min; 08:30 h), poststimulation (*t* = 60 min; 10:00 h), and postprandial (*t* = ’120 min; 12:30 h). Heart rate was measured with an Equivital EQ02 LifeMonitor (Cambridge, UK). Heart rate variability (low/high frequency) was calculated using Kubios HRV v2.1 [26].

### Clinical parameters

At both study days, the following clinical and disease activity parameters were obtained: visual analogue scale for global disease activity (VAS GDA, 0–100 mm), tender joint count of 28 joints (TJC28), swollen joint count of 28 joints (SJC28), erythrocyte sedimentation rate (ESR) in millimeters per hour, C-reactive protein (CRP) levels in milligrams per liter, and disease activity score of 28 joints (DAS28) [27]. IgM for rheumatoid factor (IgM-RF) was measured using IgM-RF Elisa from Hycor Biomedical, Indianapolis, IN (upper limit of normal (ULN) 49 IU/mL). Anti-citrullinated protein antibodies (ACPA) were measured using anti-CCP2 ELISA CCPlus (Eurodiagnostica, Nijmegen, the Netherlands (ULN 25 kAU/L)).

### Assays

Growth hormone (GH) was measured by chemiluminescence immuno-assay (Liaison, Diasorin S.p.A., Italy) with an intra-assay variation of 4.7% at 1.6 mU/L, 3.5% at 6.2 mU/L, and 3.3% at 15.0 mU/L; an inter-assay variation of 10.0% at 0.74 mU/L, 6.0% at 5.1 mU/L, and 6.0% at 18.3 mU/L; and detection limit of 0.3 mU/L.

Thyroid stimulating hormone (TSH) was measured by electro-chemiluminescence immuno-assays (ECLIA) on a Cobas E602 (Roche diagnostics). The intra-assay variation was 0.9% at 0.156 IU/L and 0.8% at 2.952 IU/L, the inter-assay variation was 1.4% at 0.156 IU/L and 1.4% at 2.952 IU/L. The detection limit was 0.01 IU/L.

ACTH was measured by luminescence enzyme immuno-assay on an Immulite 2000 (Siemens Healthcare Diagnostics B.V., Breda, the Netherlands). The intra-assay variation was 3.0–8.9%, and the inter-assay variation was 5.2–8.9%. The detection limit was 1 ng/L.

Follicle-stimulating hormone (FSH) was measured by ECLIA on a Cobas E602 (Roche diagnostics, Almere, the Netherlands), with an intra-assay variation of 1.2% at 9.7 E/L and 1.0% at 56.8 E/L, an inter-assay variation of 2.5% at 9.7 E/L and 2.5% at 56.8 E/L, and detection limit of 1 IU/L.

Luteinizing hormone (LH) was measured by ECLIA on a Cobas E602 (Roche diagnostics), with an intra-assay variation of 1.7% at 4.7 E/L and 1.6% at 52.2 E/L, an inter-assay variation of 1.6% at 4.7 IU/L and 1.6% at 52.2 E/L, and detection limit of 1 IU/L.

PRL was measured by solid-phase, two-site, time-resolved fluoro-immunometric assay (Delfia Prolactin, PerkinElmer, Turku, Finland) with an intra-assay variation of 4% at 5 μg/L and 6% at 24 μg/L, inter-assay variation of 5.5% at 4 μg/L and 7.2% at 50 μg/L, and a detection limit of 1.0 μg/L.

Cortisol was measured by luminescence enzyme immuno-assay (Siemens Healthcare Diagnostics B.V., Breda, the Netherlands). The intra-assay variation was 3.6–6.4% and the inter-assay variation was 4.7–9.0%. The detection limit was 30 nmol/L.

(Nor)epinephrine was measured with in-house high-performance liquid chromatography (HPLC). Norepinephrine and epinephrine were selectively isolated by liquid-liquid extraction and derivatized to fluorescent components with 1,2-diphenylethylenediamine. The fluorescent derivatives were separated by reversed phase liquid chromatography and detected by scanning fluorescence detection. The intra-assay variation was 6–8% for norepinephrine and 6–8% for epinephrine. The inter-assay variation was 7–12% for norepinephrine and 7–10% for epinephrine. The detection limit for both was 0.05 nmol/L.

Four different species of unconjugated bile acids (cholic acid, chenodeoxycholic acid, deoxycholic acid and ursodeoxycholic acid) and their glycine- and taurine-conjugated forms were measured using a highly sensitive HPLC tandem-MS method as previously reported [28]. Total bile acid concentrations reported are the calculated sum of all measured bile acid concentrations.

Free fatty acids (FFAs) were measured by enzymatic method (NEFAC; Wako Chemicals, Neuss, Germany). The intra-assay variation was 1% at 0.22 mmol/L and 1% at 0.93 mmol/L, and the inter-assay variation was 15% at 0.01 mmol/L and 4% at 0.48 mmol/L. The detection limit was 0.02 mmol/L.

Insulin-like growth factor-1 (IGF-1) was measured by chemiluminescence immuno-assay (Liaison, Diasorin S.p.A., Italy). The intra-assay variation is 8.0% at 10.3 nmol/L, 8.0% at 17.5 nmol/L, and 9.0% at 23.8 nmol/L. The inter-assay variation is 10% at 6.9 nmol/L, 7.4% at 30.8 nmol/L, and 8.0% at 59.4 nmol/L. The detection limit was 0.4 nmol/L.

Insulin was measured by chemiluminescent immunometric assay (Siemens Healthcare Diagnostics B.V., Breda, the Netherlands). The intra-assay variation was 6% at 47 pmol/L and 3% at 609 pmol/L. The inter-assay variation was 4% at 91 pmol/L and 6% at 120 pmol/L. The detection limit was 15 pmol/L.

C-peptide was measured with a RIA (RIA-coat C-peptide, Byk Sangtec Diagnostica, Dietzenbach, Germany). The inter-assay variation was 9% at 100 pmol/L and 7% at 500 pmol/L. The detection limit was 50 pmol/L.

Glucose was measured by spectrophotometric method (Cobas C702, Roche diagnostics, Almere, the Netherlands). The intra-assay variation was 0.8% at 5.3 mmol/L and 0.7% at 13.4 mmol/L, and the inter-assay variation was 1.3% at 5.3 mmol/L and 1.1% at 13.4 mmol/L. The detection limit was 0.1 mmol/L.

All hormone measurements, except ACTH, were performed in duplicate. Of these duplicates, means were used for the analysis.

### Statistical analysis

All subjects served as their own controls. All data were analyzed with nonparametric tests. Data are presented as median and interquartile range (IQR) or mean and standard error of the mean (SEM). AUC is calculated using the trapezoidal rule. Differences between study groups were analyzed using Wilcoxon matched pairs test where appropriate. Categorical data were analyzed using chi-square test or, if more appropriate, Fisher’s exact test. Correlations between variables were analyzed using Spearman’s rank correlation coefficient. Statistical analysis was performed using IBM SPSS Statistics 18 (SPSS Inc., Chicago, IL).

## Results

### Study population

Baseline patient characteristics are detailed in Table 2. RA disease activity, blood pressure, and heart frequency were not different between both study visits.

**Table 2:** Patient characteristics

	Female RA patients (*n* = 6)	
	Day 1: no VNS	Day 2: VNS
Age (years)	44 (38–52)	
BMI (kg/m^2^)	22.5 (21.7–22.9)	
RR systolic	115 (109–117)	118 (113–127)
RR diastolic	71 (66–75)	75 (71–80)
Heart rate	76 (70–78)	74 (73–83)
Alcohol use: *n* (%)	3 (50)	
Current smoker: *n* (%)	0 (0)	
Previous smoker: *n* (%)	1 (17)	
RF positive: *n* (%)	4 (67)	
ACPA positive: *n* (%)	2 (33)	
Disease duration (months)	130 (74–165)	
VAS GDA (mm)	33 (26–40)	35 (25–56)
TJC28 (*n*)	7 (2–12)	9 (2–12)
SJC28 (*n*)	3 (1–3)	3 (2–4)
ESR (mm/h)	13 (10–24)	8 (4–11)
CRP (mg/L)	10.1 (4.3–16.7)	4.3 (1.7–15.2)
DAS28	3.97 (3.23–4.96)	3.84 (3.28–4.83)
NSAIDs: *n* (%)	5 (83)	
Corticosteroids: *n* (%)	4 (67)	
Methotrexate: *n* (%)	6 (100)	
Adalimumab: *n* (%)	1 (17)	

Data presented as median (interquartile range) or number (percentage)

*VNS* vagus nerve stimulation, *BMI* body mass index, *RF* rheumatoid factor, *ACPA* anti-citrullinated protein antibodies, *VAS GDA* visual analogue scale (range 0–100 mm) global disease activity, *TJC28* tender joint count of 28 joints, *SJC28* swollen joint count of 28 joints, *ESR* erythrocyte sedimentation rate, *CRP* C-reactive protein, *HCQ* hydroxychloroquine, *NA* not applicable

### Hypothalamic-pituitary axis

**Fig. 1:**
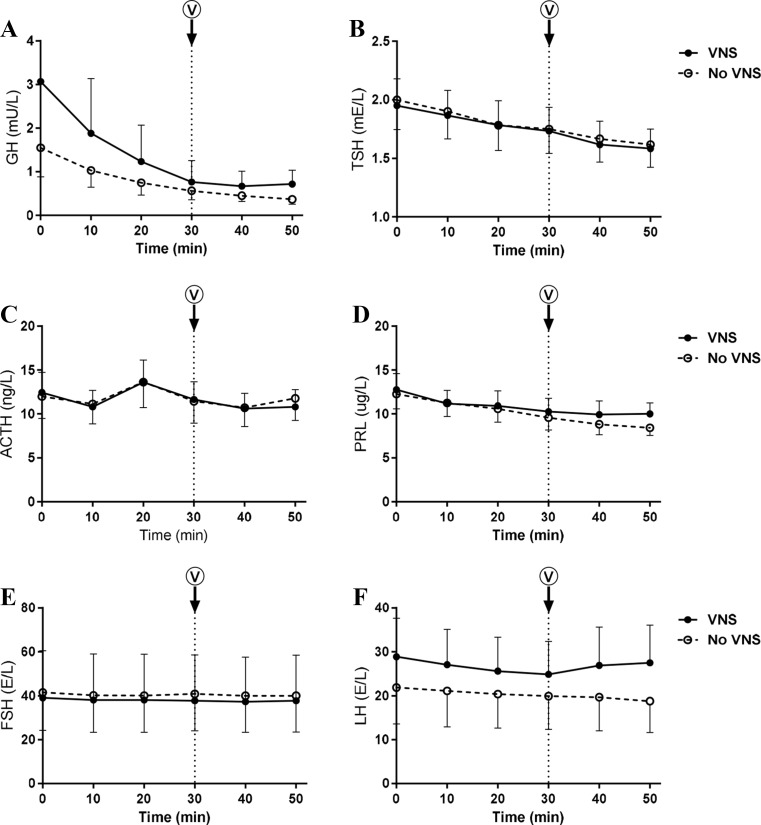
Pituitary hormones. Plasma concentrations of GH (**a**), TSH (**b**), ACTH (**c**), PRL (**d**), FSH (**e**), and LH (**f**) before and after VNS. Values are mean ± SEM. *p < 0.05

The HPA axis was evaluated by analyzing plasma levels of pituitary hormones (GH, TSH, ACTH, PRL, FSH, and LH) before and after single VNS. Baseline concentrations were comparable on study days. VNS did not affect plasma concentrations of any of the pituitary hormone measured (Fig. 1).

### SAS response and inflammation

**Fig. 2:**
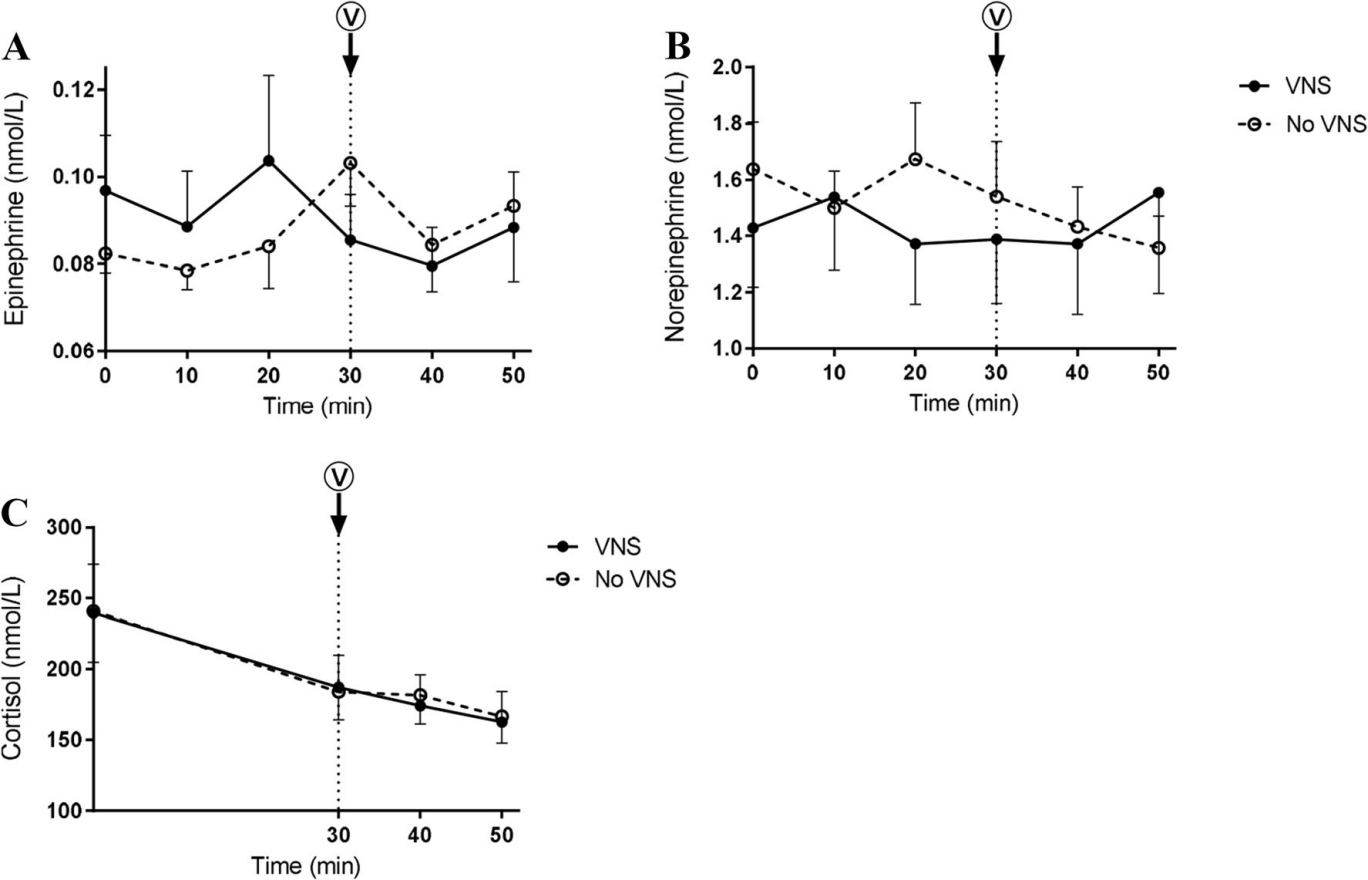
SAS response. Plasma concentrations of epinephrine (**a**), norepinephrine (**b**), and cortisol (**c**) before and after VNS. Values are mean ± SEM. *p < 0.05

Basal cortisol and catecholamine levels did not differ between study days. Neither cortisol nor catecholamines were affected by VNS (Fig. 2).

### Pre- and postprandial metabolism, EE, and HRV

**Fig. 3:**
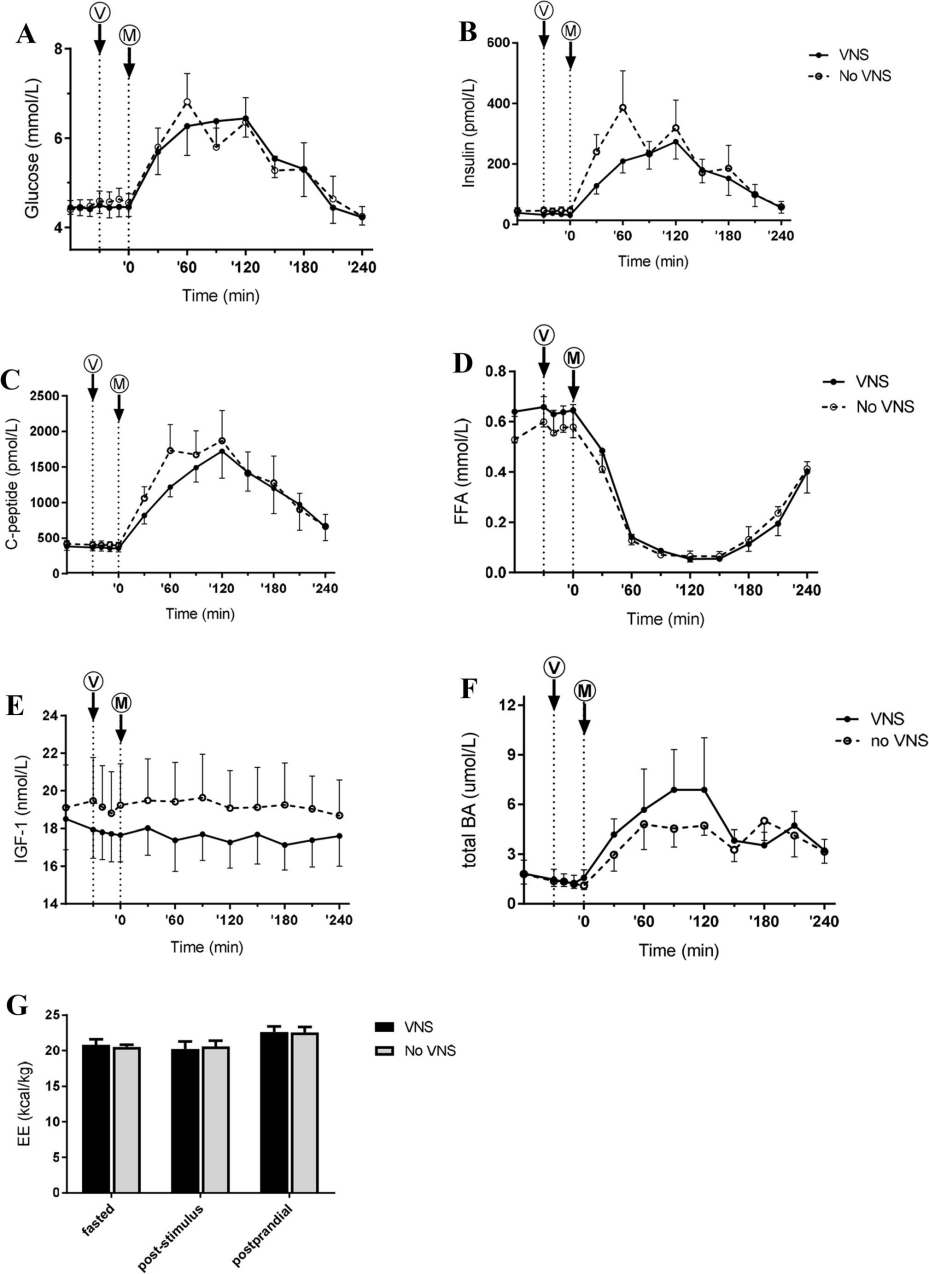
Postprandial metabolism. Plasma concentrations of glucose (**a**), insulin (**b**), C-peptide (**c**), free fatty acids (FFA) (**d**), IGF-1 (**e**), total bile acids (BA) (**f**), and BMR (**g**). *V* and *M* denote VNS and meal, respectively. Values are mean ± SEM. *p < 0.05

**Fig. 4:**
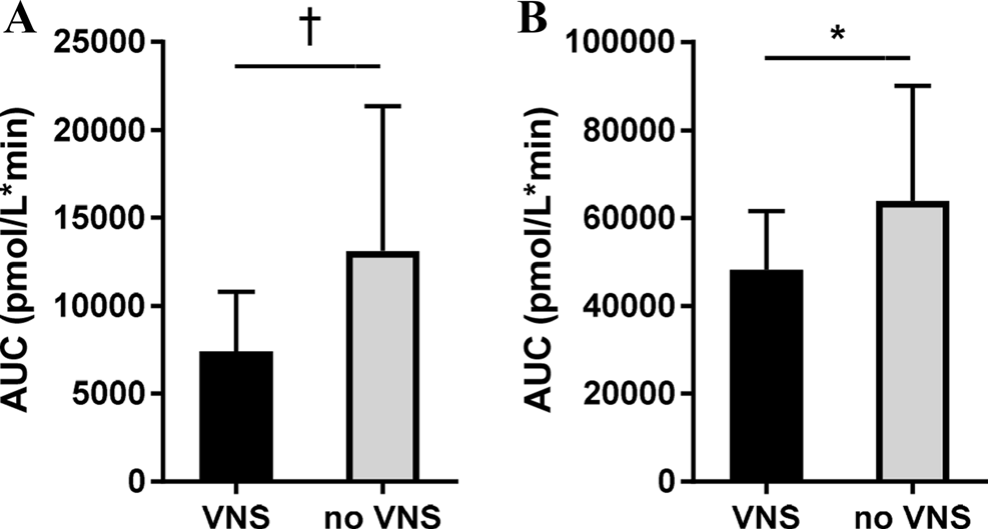
First-phase insulin and C-peptide secretion. AUC of 1-h postprandial insulin (**a**) and C-peptide (**b**) concentration. Values are mean ± SEM. ^†^ *p* < 0.10, *p < 0.05

Baseline glucose, insulin, FFA, IGF-1, and bile acid levels were comparable on study days and were not changed by VNS alone (Fig. 3). The postprandial glucose response was slightly decreased by VNS, but this did not reach statistical significance. VNS did not alter the AUC of postprandial plasma insulin levels. However, the typical early phase insulin peak was lacking after VNS. VNS tended to reduce plasma insulin levels at ’30 (*P* = 0.06). Likewise, first phase insulin secretion (plasma insulin levels AUC first 60 min after the meal) tended to be lower after VNS (*P* = 0.07) (see Fig. 4). The effect of VNS on insulin could be confirmed by measuring the C-peptide levels. VNS reduced the first phase C-peptide secretion (plasma C-peptide levels AUC first 60 min after the meal; *P* = 0.03). VNS did not affect postprandial bile acid levels.

Baseline EE was 20.8 ± 0.8 and 20.1 ± 0.5 kcal/kg on the study day with or without VNS, respectively. Poststimulus EE was 19.5 ± 1.1 and 19.5 ± 1.3 kcal/kg, respectively. The meal increased postprandial EE, but this did not differ between study days. We did not detect any effects of VNS on eardrum temperature or HRV (data not shown).

## Discussion

The results show the effect of VNS on plasma levels of hormones and other parameters of metabolic activity in a cohort of patients with RA treated with VNS. Overall, a single VNS reduced the first phase C-peptide secretion but did not affect these parameters. This suggests that acute VNS treatment in RA patients has no major interactions with other vagal targets and supports the concept that VNS is a treatment without major effects on endocrinologic and metabolic parameters.

VNS did not alter plasma levels of cortisol and catecholamine levels in this study. This is in line with animal studies, in which acute VNS had no effect on either the HPA axis or the sympathetic-adrenal system (SAS) response [15]. However, there is some evidence that chronic VNS might facilitate the recovery of the stress-induced HPA axis response via effective stress coping strategies, e.g., more efficient negative feedback following a stressor. A study in rats documented that continuous VNS during 3 h increases norepinephrine concentrations in the brain [16]. Others have examined a group of patients with chronic depression and observed a reduction in the ACTH response upon CRH challenge after 2 weeks of VNS treatment [17]. Thus, although long-term VNS may have effects on the HPA axis and/or SAS response, this is not the case for single VNS as shown in this study.

We also observed that VNS did not affect plasma levels of other pituitary hormones. The evidence on interaction between vagal activity and pituitary hormone levels in plasma so far is conflicting. In one report, bilateral vagotomy in rats resulted in a significant increase in plasma PRL levels after 7 days [14]. In another study, the suckling-induced increase of prolactin in vagotomized lactating rats was significantly decreased [18]. Interestingly, it has been suggested in the literature that PRL interacts with the immune system, providing a possible additional pathway for VNS effects on inflammation [29–31]. However, we did not observe any effects of VNS on PRL levels. In another study in rats, disruption of the vagus nerve caused lower GH levels [32].

VNS did not affect postprandial curves of glucose, insulin, C-peptide, IGF-1, fatty acid, and bile acid levels either before or after the meal, although the curves of glucose, insulin, C-peptide, and bile acids suggested a possible VNS effect (Fig. 4). Recently, it has been suggested that efferent VNS might be potentially effective in treating type II diabetes [19]. In contrast to our findings, in Zucker diabetic rats [27], transauricular VNS decreased fasting glycemia and body weight increase when applied over a longer time frame. In the only trial performed in humans, transauricular VNS treatment increased glucose tolerance in impaired glucose tolerant patients over 12 weeks, though the effect was very small compared to sham treatment. Conversely, in controls, but not in vagotomized participants, intravenous glucose administration significantly inhibits pancreatic polypeptide secretion suggesting a vagal glucose sensing mechanism dependent on intact vagal innervation [33].

The link between VNS and plasma insulin levels is less clear, although vagal activity has been implicated in insulin secretion. During the first hour after the meal, VNS clearly showed reduced insulin secretion as evidenced from lower plasma C-peptide levels. Hyperinsulinemia is present in animal models of obesity and type 2 diabetes that is thought to be associated with parasympathetic drive to the pancreatic ß-cells [34–38]. Most of these experimental studies used indirect approaches to modulate vagal activity. Direct vagal stimulation effects on insulin secretion have not been investigated. However, the early pre-absorptive insulin response to meal ingestion in humans has been attributed to autonomic activation by noncholinergic and cholinergic mechanisms [39]. This is of interest with respect to the significant lower C-peptide levels and trend for lower plasma insulin levels. C-peptide is not cleared by the liver and other organs, and thus reflects endogenous insulin secretion more accurately than insulin [40, 41].

We analyzed baseline and postprandial bile acid concentrations since bile acid homeostasis has a circadian rhythm in both rodents and humans [39, 42, 43]. Bile acids are synthesized in the liver, enter the enterohepatic cycle, and are under control of the nuclear farnesoid X receptor [44]. Older studies suggest that bile acid secretion may be under vagal control [45, 46]. However, in this acute VNS setting, we could not support this notion.

We found no effect on EE or eardrum temperature in contrast to previous work [21] that suggested a marginal 2.2% increase in EE in epileptic patients stimulated every 5 min, performing indirect calorimetry over the course of 2 h. This discrepancy can be explained by our single bout of VNS compared to more frequent stimulation in epilepsy. Of note, RA patients received up to a maximum of 60 s stimulation eight times a day [22]. VNS patients describe the more frequent stimulation as mildly unpleasant, and repetitive bouts could perhaps increase a cortisol/catecholamine response which was not seen in our study.

This is the first study in which plasma levels of pituitary hormones, catecholamines, metabolic activity, and postprandial metabolism have been determined systemically in response to VNS. A limitation of the study is the small number of study patients. This is still a highly novel and experimental approach in RA, and only 17 RA patients have been treated with VNS so far [22]. Since we excluded pre-existent pituitary disease or metabolic disorders (i.e., diabetes mellitus), six was the maximum number of eligible patients. To strengthen the robustness of data, the results were built on paired data for all the study patients. Another caveat is that the included patients had already been treated with VNS for at least 3 months; thus, effects of chronic VNS may have obscured acute effects that would be seen in VNS-naïve individuals. Although patients were instructed not to employ VNS 24 h before study days, we cannot completely exclude the possibility that this has not been long enough. However, the strengths of our study are the crossover design and the systematic measurements of the hormones and other parameters of metabolic activity on consecutive timepoints. Still, we would have expected an immediate effect of VNS on hormone levels in light of the infrequent dosing regimen.

VNS is a potentially promising and transformational therapy for patients with inflammatory diseases as recently suggested based on the first clinical trial in patients with RA [22]. Previous animal and human studies suggest that the vagus nerve plays a role in neuroendocrine adaptation and therefore VNS could have affected metabolic adaptation. We did not find any evidence for this notion if VNS is used up to eight times a day, except perhaps for insulin secretion. Further studies are needed to determine the effects of different VNS durations, frequencies, and intensities. Apart from insulin secretion, single VNS in subjects with RA that have been treated with VNS at least 3 months has no discernible acute effects on hormone levels or metabolism. Therefore, this suggests VNS as a safe and effective treatment for patients suffering from rheumatoid arthritis. Furthermore, the possible and favorable effect on insulin secretion needs to be investigated.

## References

[1] Steinman L (2004). Elaborate interactions between the immune and nervous systems. Nat Immunol.

[2] Koopman FA, Stoof SP, Straub RH, Van Maanen MA, Vervoordeldonk MJ, Tak PP (2011). Restoring the balance of the autonomic nervous system as an innovative approach to the treatment of rheumatoid arthritis. Mol Med.

[3] Berthoud HR, Neuhuber WL (2000). Functional and chemical anatomy of the afferent vagal system. Auton Neurosci.

[4] Barraco R, el-Ridi M, Ergene E, Parizon M, Bradley D (1992). An atlas of the rat subpostremal nucleus tractus solitarius. Brain Res Bull.

[5] Tracey KJ (2009). Reflex control of immunity. Nat Rev Immunol.

[6] Koopman FA, Tang MW, Vermeij J, de Hair MJ, Choi IY, Vervoordeldonk MJ, Gerlag DM, Karemaker JM, Tak PP (2016). Autonomic dysfunction precedes development of rheumatoid arthritis: a prospective cohort study. EBioMedicine.

[7] van Maanen MA, Vervoordeldonk MJ, Tak PP (2009). The cholinergic anti-inflammatory pathway: towards innovative treatment of rheumatoid arthritis. Nat Rev Rheumatol.

[8] Bonaz B, Sinniger V, Hoffmann D, Clarencon D, Mathieu N, Dantzer C, Vercueil L, Picq C, Trocme C, Faure P, Cracowski JL, Pellissier S (2016). Chronic vagus nerve stimulation in Crohn’s disease: a 6-month follow-up pilot study. Neurogastroenterol Motil.

[9] Tang MW, Koopman FA, Visscher JP, de Hair MJ, Gerlag DM, Tak PP (2016). Hormone, metabolic peptide, and nutrient levels in the earliest phases of rheumatoid arthritis—contribution of free fatty acids to an increased cardiovascular risk during very early disease. Clin Rheumatol.

[10] Beekwilder JP, Beems T (2010). Overview of the clinical applications of vagus nerve stimulation. J Clin Neurophysiol.

[11] Koopman FA, Schuurman PR, Vervoordeldonk MJ, Tak PP (2014). Vagus nerve stimulation: a new bioelectronics approach to treat rheumatoid arthritis?. Best Pract Res Clin Rheumatol.

[12] Koopman FA, Chavan SS, Miljko S, Grazio S, Sokolovic S, Schuurman PR, Mehta AD, Levine YA, Faltys M, Zitnik R, Tracey KJ, Tak PP (2016). Vagus nerve stimulation inhibits cytokine production and attenuates disease severity in rheumatoid arthritis. Proc Natl Acad Sci U S A.

[13] Gerendai I, Drago F, Continella G, Scapagnini U (1984). Effects of mastectomy and vagotomy on grooming behavior of the rat: possible involvement of prolactin. Physiol Behav.

[14] Eriksson M, Bjorkstrand E, Smedh U, Alster P, Matthiesen AS, Uvnas-Moberg K (1994). Role of vagal nerve activity during suckling. Effects on plasma levels of oxytocin, prolactin, VIP, somatostatin, insulin, glucagon, glucose and of milk secretion in lactating rats. Acta Physiol Scand.

[15] Thrivikraman KV, Zejnelovic F, Bonsall RW, Owens MJ (2013). Neuroendocrine homeostasis after vagus nerve stimulation in rats. Psychoneuroendocrinology.

[16] Follesa P, Biggio F, Gorini G, Caria S, Talani G, Dazzi L, Puligheddu M, Marrosu F, Biggio G (2007). Vagus nerve stimulation increases norepinephrine concentration and the gene expression of BDNF and bFGF in the rat brain. Brain Res.

[17] O’Keane V, Dinan TG, Scott L, Corcoran C (2005). Changes in hypothalamic-pituitary-adrenal axis measures after vagus nerve stimulation therapy in chronic depression. Biol Psychiatry.

[18] Szekely M (2000). The vagus nerve in thermoregulation and energy metabolism. Auton Neurosci.

[19] Meyers EE, Kronemberger A, Lira V, Rahmouni K, Stauss HM (2016) Contrasting effects of afferent and efferent vagal nerve stimulation on insulin secretion and blood glucose regulation. Physiol Rep 4(4). doi:10.14814/phy2.12718 10.14814/phy2.12718 PMC4759047 26884478

[20] Vijgen GH, Bouvy ND, Leenen L, Rijkers K, Cornips E, Majoie M, Brans B, van Marken Lichtenbelt WD (2013). Vagus nerve stimulation increases energy expenditure: relation to brown adipose tissue activity. PLoS One.

[21] Adlan AM, Lip GY, Paton JF, Kitas GD, Fisher JP (2014). Autonomic function and rheumatoid arthritis—a systematic review. Semin Arthritis Rheum.

[22] Koopman FA, Chavan SS, Miljko S, Grazio S, Sokolovic S, Schuurman PR, Mehta AD, Levine YA, Faltys M, Zitnik R, Tracey KJ, Tak PP (2016). Vagus nerve stimulation inhibits cytokine production and attenuates disease severity in rheumatoid arthritis. Proc Natl Acad Sci U S A.

[23] Aletaha D, Neogi T, Silman AJ, Funovits J, Felson DT, Bingham CO III, Birnbaum NS, Burmester GR, Bykerk VP, Cohen MD, Combe B, Costenbader KH, Dougados M, Emery P, Ferraccioli G, Hazes JM, Hobbs K, Huizinga TW, Kavanaugh A, Kay J, Kvien TK, Laing T, Mease P, Menard HA, Moreland LW, Naden RL, Pincus T, Smolen JS, Stanislawska-Biernat E, Symmons D, Tak PP, Upchurch KS, Vencovsky J, Wolfe F, Hawker G (2010) 2010 Rheumatoid arthritis classification criteria: an American College of Rheumatology/European League Against Rheumatism collaborative initiative. Arthritis Rheum 62(9):2569–2581. doi:10.1002/art.27584%20%5B%5D 10.1002/art.27584 20872595

[24] Neogi T, Aletaha D, Silman AJ, Naden RL, Felson DT, Aggarwal R, Bingham CO, Birnbaum NS, Burmester GR, Bykerk VP, Cohen MD, Combe B, Costenbader KH, Dougados M, Emery P, Ferraccioli G, Hazes JM, Hobbs K, Huizinga TW, Kavanaugh A, Kay J, Khanna D, Kvien TK, Laing T, Liao K, Mease P, Menard HA, Moreland LW, Nair R, Pincus T, Ringold S, Smolen JS, Stanislawska-Biernat E, Symmons D, Tak PP, Upchurch KS, Vencovsky J, Wolfe F, Hawker G (2010). The 2010 American College of Rheumatology/European League Against Rheumatism classification criteria for rheumatoid arthritis: phase 2 methodological report. Arthritis Rheum.

[25] Frayn KN (1983). Calculation of substrate oxidation rates in vivo from gaseous exchange. J Appl Physiol Respir Environ Exerc Physiol.

[26] Tarvainen MP, Niskanen JP, Lipponen JA, Ranta-Aho PO, Karjalainen PA (2014). Kubios HRV—heart rate variability analysis software. Comput Methods Prog Biomed.

[27] Anderson J, Caplan L, Yazdany J, Robbins ML, Neogi T, Michaud K, Saag KG, O'Dell JR, Kazi S (2012). Rheumatoid arthritis disease activity measures: American College of Rheumatology recommendations for use in clinical practice. Arthritis Care Res (Hoboken).

[28] Bootsma AH, Overmars H, van Rooij A, van Lint AE, Wanders RJ, van Gennip AH, Vreken P (1999). Rapid analysis of conjugated bile acids in plasma using electrospray tandem mass spectrometry: application for selective screening of peroxisomal disorders. J Inherit Metab Dis.

[29] Mathis D, Shoelson SE (2011). Immunometabolism: an emerging frontier. Nat Rev Immunol.

[30] Ouchi N, Parker JL, Lugus JJ, Walsh K (2011). Adipokines in inflammation and metabolic disease. Nat Rev Immunol.

[31] Tang MW, Reedquist KA, Garcia S, Fernandez BM, Codullo V, Vieira-Sousa E, Goffin V, Reuwer AQ, Twickler MT, Gerlag DM, Tak PP (2016). The prolactin receptor is expressed in rheumatoid arthritis and psoriatic arthritis synovial tissue and contributes to macrophage activation. Rheumatology (Oxford).

[32] Al-Massadi O, Trujillo ML, Senaris R, Pardo M, Castelao C, Casanueva FF, Seoane LM (2011). The vagus nerve as a regulator of growth hormone secretion. Regul Pept.

[33] Veedfald S, Plamboeck A, Hartmann B, Svendsen LB, Vilsboll T, Knop FK, Holst JJ (2015). Pancreatic polypeptide responses to isoglycemic oral and intravenous glucose in humans with and without intact vagal innervation. Peptides.

[34] Fukudo S, Virnelli S, Kuhn CM, Cochrane C, Feinglos MN, Surwit RS (1989). Muscarinic stimulation and antagonism and glucoregulation in nondiabetic and obese hyperglycemic mice. Diabetes.

[35] Fletcher JM, McKenzie N (1988). The parasympathetic nervous system and glucocorticoid-mediated hyperinsulinaemia in the genetically obese (fa/fa) Zucker rat. J Endocrinol.

[36] Rohner-Jeanrenaud F, Ionescu E, Jeanrenaud B (1983). The origins and role of efferent vagal nuclei in hyperinsulinemia in hypothalamic and genetically obese rodents. J Auton Nerv Syst.

[37] Sainsbury A, Rohner-Jeanrenaud F, Cusin I, Zakrzewska KE, Halban PA, Gaillard RC, Jeanrenaud B (1997). Chronic central neuropeptide Y infusion in normal rats: status of the hypothalamo-pituitary-adrenal axis, and vagal mediation of hyperinsulinaemia. Diabetologia.

[38] Berthoud HR, Jeanrenaud B (1979). Acute hyperinsulinemia and its reversal by vagotomy after lesions of the ventromedial hypothalamus in anesthetized rats. Endocrinology.

[39] Ahren B, Holst JJ (2001). The cephalic insulin response to meal ingestion in humans is dependent on both cholinergic and noncholinergic mechanisms and is important for postprandial glycemia. Diabetes.

[40] Saisho Y (2016) Postprandial C-peptide to glucose ratio as a marker of beta cell function: implication for the management of type 2 diabetes. Int J Mol Sci 17(5). doi:10.3390/ijms17050744 10.3390/ijms17050744 PMC4881566 27196896

[41] Polonsky KS, Rubenstein AH (1984). C-peptide as a measure of the secretion and hepatic extraction of insulin. Pitfalls and limitations. Diabetes.

[42] Galman C, Angelin B, Rudling M (2005). Bile acid synthesis in humans has a rapid diurnal variation that is asynchronous with cholesterol synthesis. Gastroenterology.

[43] Back P, Hamprecht B, Lynen F (1969). Regulation of cholesterol biosynthesis in rat liver: diurnal changes of activity and influence of bile acids. Arch Biochem Biophys.

[44] Lefebvre P, Cariou B, Lien F, Kuipers F, Staels B (2009). Role of bile acids and bile acid receptors in metabolic regulation. Physiol Rev.

[45] Smith RB, Edwards JP, Johnston D (1981). Effect of vagotomy on exocrine pancreatic and biliary secretion in man. Am J Surg.

[46] Wisen O, Rossner S, Johansson C (1988). Impaired pancreatico-biliary response to vagal stimulation and to cholecystokinin in human obesity. Metabolism.

